# Topical Administration of Terpenes Encapsulated in Nanostructured Lipid-Based Systems

**DOI:** 10.3390/molecules25235758

**Published:** 2020-12-07

**Authors:** Elwira Lasoń

**Affiliations:** Faculty of Chemical Engineering and Technology, Cracow University of Technology, Warszawska St 24, 31-155 Kraków, Poland; elwira.lason@pk.edu.pl; Tel.: +48-12-628-2761

**Keywords:** terpenes, terpenoids, lipid nanoparticles, nanostructured lipid carriers, topical administration, lipid-based systems

## Abstract

Terpenes are a group of phytocompounds that have been used in medicine for decades owing to their significant role in human health. So far, they have been examined for therapeutic purposes as antibacterial, anti-inflammatory, antitumoral agents, and the clinical potential of this class of compounds has been increasing continuously as a source of pharmacologically interesting agents also in relation to topical administration. Major difficulties in achieving sustained delivery of terpenes to the skin are connected with their low solubility and stability, as well as poor cell penetration. In order to overcome these disadvantages, new delivery technologies based on nanostructures are proposed to improve bioavailability and allow controlled release. This review highlights the potential properties of terpenes loaded in several types of lipid-based nanocarriers (liposomes, solid lipid nanoparticles, and nanostructured lipid carriers) used to overcome free terpenes’ form limitations and potentiate their therapeutic properties for topical administration.

## 1. Introduction

Terpenes and their oxygenated derivatives terpenoids are the largest and most common class of secondary metabolites. Terpenoids are modified terpenes where methyl groups are moved or removed, or additional functional groups (usually oxygen-containing) are added. The two terms are often used interchangeably. They are found in higher plants, as representing the majority of molecules in essential oils, mosses, liverworts, and algae lichens, as well as insects, microbes, and marine organisms [1,2,3]. These compounds are a very diverse group of molecules with an extremely varied structure and function [4]. The basic chemical structure of terpenes and terpenoids contains several repeated isoprene units (C_5_H_8_) used to classify them. Thus, e.g., hemiterpenes (hemiterpenoids) are formed by one isoprene unit, monoterpenes (monoterpenoids) have two isoprene units (C10), sesquiterpenes (sesquiterpenoids) have three (C15), and diterpenes (diterpenoids) have four (C20) isoprene units. Terpenes may also be classified as linear, monocyclic, and bicyclic [3,5]. The volatility of these compounds decreases with an increased number of isoprene units [6]. Smaller terpenes are not only highly volatile but also susceptible to degradation mainly by oxidation and isomerization and usually thermolabile [5,7].

Targeted delivery of typically hydrophobic terpenoids has been the subject of much research, finding applications in a wide variety of fields. As naturally occurring compounds, they have been applied in transdermal research since the 1960s and are reported to be a safe and effective class of penetration enhancers [8,9]. Plenty of them have been used as antispasmodics, carminatives, flavoring agents, or perfumes [3]. Several studies also indicated that terpenoids can suppress nuclear factor-κB (NF-κB) signaling, the major regulator in the pathogenesis of inflammatory diseases and cancer [10], thus confirming anti-inflammatory [11] and antineoplastic applications [12,13]. Additionally, terpenes were also found as cutaneous wound healing accelerators [14]. Because that class of compounds appears to offer great benefits for therapeutic purposes, it is understood that the proper carriers play an important role in their administration. Therefore, efficient, safe, and natural delivery systems are of great interest.

Lipid-based nanocarriers are novel drug delivery systems that have been widely explored for topical and transdermal delivery of pharmaceuticals. Encapsulation of terpenoids in such a system is an interesting strategy to provide better stability and protection against environmental factors that may cause chemical degradation. In addition, nanoencapsulation can decrease the toxicity, solubilize poorly soluble terpenes, improve bioavailability, and achieve controlled and sustained delivery, in addition to drug targeting at the site of action [15,16].

In the present review, the topical administration of selected terpenoids is discussed with special emphasis on nanostructured delivery systems applied as carriers for these groups of bioactive compounds.

## 2. Topical Route of Terpenes Administration

The topical administration of bioactive compounds acting as drugs relies on the localized administration of formulations to a body through dermal and mucosal (e.g., ocular, vaginal, nasal, and rectal) routes. Skin is one of the most easily accessible organs in the human body for topical administration and is the major route of localized drug delivery [5,17]. The drug delivery system can be considered dermal when the targeting site of the drug is the skin or transdermal when the drug has to pass through skin layers to reach the target and, by analogy, for mucosal tissue administration, delivery can be mucosal and transmucosal [18].

**Figure 1:**
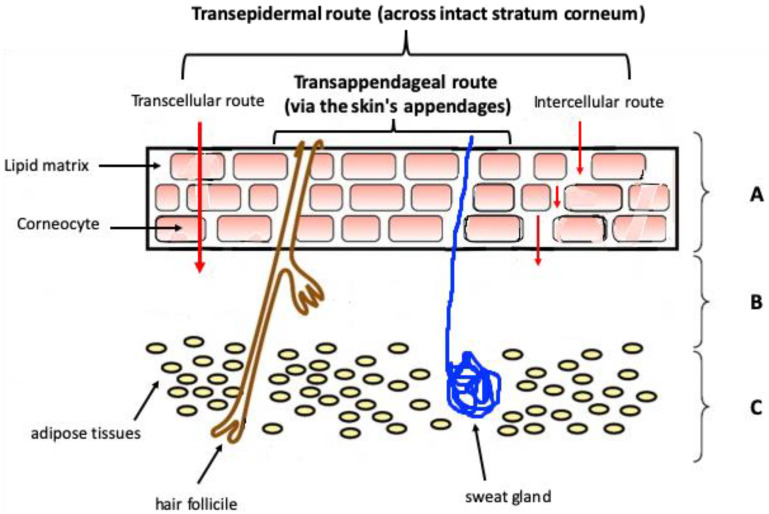
Diagrammatic illustration of the skin’s structure and routes of permeation through the skin. A—Epidermis (SC), B—Dermis, C—Subcutaneous layer.

The intact skin is much less permeable than other tissues and penetration of the active compounds depends on the physicochemical properties of the penetrant, the condition of the skin, and the nature of the carrier [17]. Topically applied drugs may diffuse through the skin by hair follicles, sweat glands, or sebaceous glands, but the predominant and very slow route is through multiple lipid bilayers of the stratum corneum (Figure 1). Terpenes can be applied topically mainly for local action, e.g., as wound healing, antiseptic, anti-fungal, or anti-inflammatory agents but at the same time, this route can be used to reach the deeper layer of the skin or even for systemic drug delivery like in case of anesthetic or antihypertensive acting terpenes.

Drug administration through mucosal and transmucosal routes like oral, nasal, and ocular is still a challenge for scientists mainly because of the presence of mucus, saliva, and lacrimal fluids, which can restrict the access of bacteria or virus to the deeper layers but can also affect the bioavailability of bioactive compounds like terpenes [18,19].

Topical terpenoids application can be an attractive route of administration as it allows the reduction of side effects, avoids the injectable route, the first-pass metabolism, and pre-systemic elimination within the gastrointestinal tract. Moreover, it is a safe route with the easiness of application and fast action [20].

### 2.1. Terpenes as Skin Permeation Enhancers

The effectiveness of transdermal drug delivery depends on the sufficient capability of drugs to penetrate through the skin to reach the therapeutic level. An important barrier of the skin for drug absorption is the stratum corneum (SC) [21,22]. The stratum corneum consists of keratin-enriched dead cells, surrounded by crystalline intercellular lipid lamellar structures. These are continuous structures in the SC and are required for competent skin barrier function [3]. To facilitate drug delivery through the skin and increase percutaneous absorption, penetration enhancers are extensively used, as they ideally cause a temporary reversible reduction in the barrier function of the SC [9].

Terpenes and terpenoids have attracted great interest as effective enhancers from natural products [3,23,24,25,26]. They are commonly considered to be less toxic with low irritancy potential compared to other synthetic skin penetration enhancers or surfactants. Moreover, this class of penetration enhancers has been classified by the Food and Drug Administration (FDA) as generally recognized as safe (GRAS) agents [8,24]. Terpenes can increase skin permeation by one or more mechanisms, including interaction with SC lipids and/or keratin and increasing the solubility of a drug into SC lipids. Nevertheless, the interaction of terpenes with SC in the presence of various solvents may not be similar due to differences in the physicochemical properties of these solvents and their interactions with SC, but there are some instrumental methods (e.g., differential scanning calorimetry (DSC) and Fourier transform infrared spectroscopy (FTIR)), which can help to determine these interactions [24].

**Figure 2:**
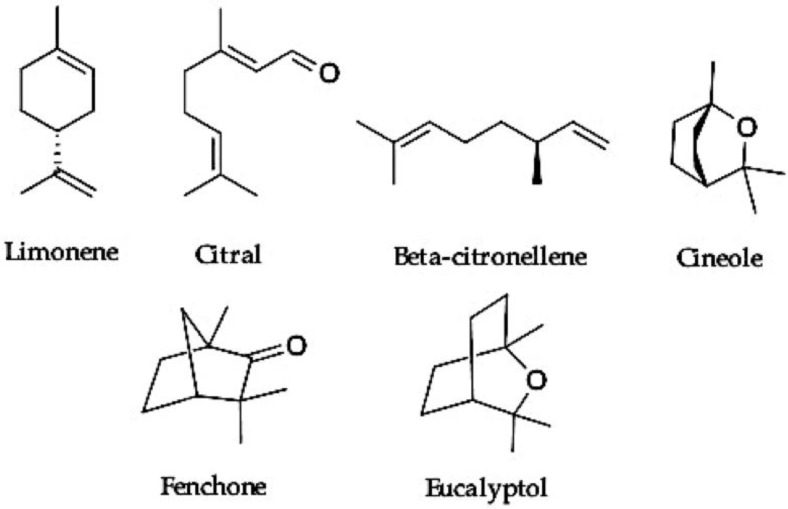
Representatives of monoterpenes and monoterpenoids applied as skin permeation enhancers.

Terpenes acting as permeation enhancers are usually used as excipients in formulations (Table 1) and are capable of facilitating the passage of the main drug through the skin. The nanostructured lipid-based systems in which terpenes are used as enhancers are mainly invasomes, liposomes, nanoemulsions, and SLN (Solid Lipid Nanoparticles)/NLC (Nanostructured Lipid Carriers) [5]. Invasomes are composed of phosphatidylcholine, ethanol, and a mixture of terpenes and are most popular in recent years among the formulations of nanosystems using terpenes as excipients [27,28]. They present elasticity and deformability, which favors penetration across skin layers, and thus they work as penetration-enhancing vesicles [29]. Terpenes applied in the formulations of invasomes or other lipid-based carriers are mainly representatives of monoterpenes and monoterpenoids (Figure 2) [27,28,29,30,31,32,33,34,35,36,37,38].

### 2.2. Terpenes as Bioactive Compounds

The most widely investigated therapeutic potential of terpenes and terpenoids for topical administration is the anti-inflammatory activity, including burns or wounds healing [10,11,12,13,14].

Inflammation is the physiological response of the body to tissue injury (e.g., stress, irritants, radiations), microbial and viral infections, or genetic changes and may have an acute or chronic state [39]. The most common signs of acute inflammation are swelling, pain, erythema, and increased heat. Some chronic diseases can be developed due to inflammation and if the condition causing the damage is not resolved, the inflammatory process evolves toward subacute or chronic inflammation [11]. The chronic state of inflammation has important roles at the beginning of various diseases, including cardiovascular disease, diabetes, cancer, obesity, and asthma, as well as classic inflammatory diseases like arthritis or psoriasis [40,41,42]. Some chronic diseases may involve the use of anti-inflammatory agents such as steroidal and non-steroidal drugs, but some of them can cause undesirable side effects [11]. Therefore, there is a need to find new therapeutic alternatives that are less toxic, and the perfect candidates seem to be terpenes (Table 2).

An equally important therapeutic aspect of terpenes is their activity against skin cancer. The number of skin cancer cases has increased rapidly worldwide. Skin cancer, including melanoma and non-melanoma skin cancer (NMSC), represents the most common type of malignancy in the white population [53,54]. The major risk factor of developing cutaneous cancers is chronic exposure of the skin to UV radiation, both natural and artificial [55]. Potential risks for cancer are also genetic predisposition, depressed immune system, or exposure to viral infections (human papillomavirus), or chemicals like aromatic hydrocarbons and arsenic [54]. The huge impact on the prognosis of any type of skin cancer is its early diagnosis and immediate treatment. When it comes to melanoma skin cancer, it has a high propensity for metastasis; therefore, it is more of a systemic disease as far as treatment options are considered. For NMSC, the treatment mainly depends on the number, thickness, and distribution of lesions, then patient preferences like convenience, tolerance, and treatment cost are also taken into consideration [56]. Topical therapies are mainly applied when there are multiple lesions, the affected area is large, or for lesions that take time to cure. They are also used for patients who are not candidates for surgery [54]. Nanostructured systems hold great promise as carriers for skin cancer treatment in topical application. Numerous nanomaterials can be applied as drug vesicles from those based on lipids to polymer micelles, silicone dioxide, carbon nanotubes, gold, silver, and other metal or metal oxides [57,58]. As drug carriers, nanoparticles must have low toxicity and deliver drugs precisely into target tissues in order to achieve the maximum benefit with minimum side effects [59]. The nanocarriers’ ability to treat tumors has been extensively investigated by many research groups [54,59,60,61]. For decades, lipid-based nanoparticle systems have been tested in vitro and in vivo for the topical treatment of skin cancer mainly because of their ability to improve skin and tumor penetration of bioactive compounds. The therapeutic potential of terpenoids and terpenes as antineoplastic drugs is also well known as that which gives promising perspectives in topical skin cancer treatment (Table 3).

## 3. Lipid-Based Nanoparticles for Topical Applications of Terpenes

**Figure 3:**
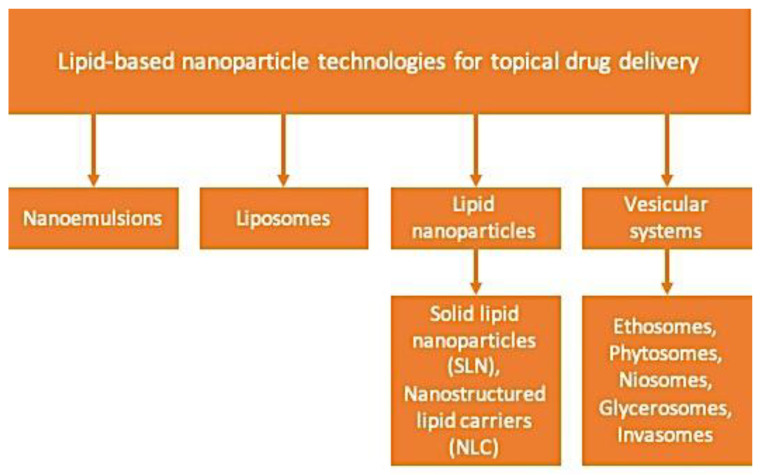
Nanoparticle based on lipids technologies for topical drug delivery.

The choice of vehicle or delivery system in the case of skin diseases has a significant influence on the outcome of topical dermatological drug treatment. Nanoparticulate systems can improve the stability of actives in front of possible degradation by light, heat, and other environmental factors. Moreover, they provide better bioavailability, improve permeation through the skin and other biological barriers, and reach the controlled delivery of drugs [16,66]. These systems can be divided according to their composition on polymer and lipid-based carriers. The lipid-based systems are formed by lipids and include nanoemulsions (NE), liposomes (LS), solid lipid nanoparticles (SLN), nanostructured lipid carriers (NLC), and vesicular systems (VS), which comprehend ethosomes, phytosomes, niosomes, glycerosomes, and invasomes (IV) (Figure 3) [66]. The examples of using different types of the vesicles mentioned above to incorporate and deliver terpenes to the skin are presented in Table 1, Table 2 and Table 3. A brief overview of some of the most commonly tested lipid systems in topical delivery is introduced below.

Nanoemulsions (NE) are thermodynamically stable oil in water (o/w) or water in oil (w/o) dispersions stabilized by surfactant molecules. The mean droplet size, ranging from 20 to 200 nm, and the low percentage of surfactant make them ideal for topical drug delivery with reduced skin irritation [67]. NEs are able to solubilize lipophilic drugs at high loading capacity. Their large surface area makes it possible to create close occlusive contact with the stratum corneum that helps to permeate and deliver drugs (both, lipo- and hydrophilic) deep inside the skin. NE skin permeation is also enhanced by the presence of oil and surfactants in the composition that may change the lipid structure of the stratum corneum [67,68].

Liposomes are vesicles built by amphiphilic lipids, mainly cholesterol and phospholipids. The lipids arrange themselves in bilayers surrounding an aqueous core, where hydrophilic compounds can be entrapped while hydrophobic drugs can be encapsulated on the bilayer. Liposomes are very versatile drug delivery systems since they can incorporate either hydrophilic, hydrophobic, or amphiphilic drugs [66,69]. Liposomes have the ability to improve the pharmacokinetics, specificity, and efficacy of a drug with reduced toxicity [70]. The idea of using liposomes for skin diseases was first proposed by Mezei and Gulasekharam in 1980 [71]. The composition of the liposomes enables their adsorption on the skin surface and fusion with SC lipids, hence initiating the release of the drug into the tissue [72]. It has been shown that liposomes can accumulate in various layers of the skin compared to the free drug, so they can be engineered to achieve a desirable layer. Larger particles remain adsorbed on the surface while liposomes with a mean diameter of less than 50 nm have been shown to accumulate within the deeper layers of the tissue [73].

Lipid nanoparticles are innovative nanocarriers known as solid lipid nanoparticles (SLN) and nanostructured lipid carriers (NLC) and represent a revolution in the efficient encapsulation of hydrophobic drugs and long-term physicochemical stability of lipid-based drug delivery systems [18]. They are highly tolerable and protect drugs from degradation while maintaining a steady release for extended periods [54]. SLNs are colloidal systems consisting of a blend of biodegradable and biocompatible solid lipids, emulsifiers, and water and range from less than 50 to 1000 nm in size. Typically used lipids include triglycerides, glycerides, fatty acids, and waxes [74]. SLNs adhere to the skin to form a monolayer that creates an occlusive effect to increase the water retention of the skin. This helps to increase the penetration of drug-loaded particles into the skin [75]. Unfortunately, these systems possess some disadvantages associated with poor drug loading due to a compact lipid matrix and possible active expulsion during storage connected with matrix polymorphic transition. Nevertheless, as a result of the solid nature of SLN, they have enhanced physical stability over nanoemulsions [76]. NLCs were introduced to overcome some drawbacks represent by SLNs. They are composed of a blend of solid and liquid lipids that do not possess the ideal crystalline structure. The lipid is either enclosed within the solid lipid matrix or localized on the surfactant layer [77], which allows it to increase drug loading capacity and improve bioavailability.

Vesicular systems have been among the most studied for topical therapies during recent years [54]. The physicochemical properties of these systems allow the creation of easy-to-produce nano-scaled drug transporters. It is possible to encapsulate either polar compounds, in the inner aqueous compartment of the vesicle, or non-polar molecules, embedded in the membrane [78]. Depending on its membrane composition, vesicular systems are often classified as ethosomes, phytosomes, niosomes, glycerosomes, and invasomes [5]. Many researchers have also classified liposomes into this group due to the resemblance to the structure and composition.

Niosomes are a form of liposomes composed of nonionic surfactants that produce more stable, less toxic, and more flexible vesicles. They are also less expensive and more economical to manufacture [79]. Niosomes can modify the SC barrier by blending with the lipids. They can also increase the smoothness of the SC by recovering the lost lipids and reducing the transepidermal water loss. All these depend on the physicochemical properties of the drug, the vesicle, and the lipids used to produce the niosomes [79,80].

Ethosomes are elastic vesicles composed of phospholipids, cholesterol, water, and large amounts of ethanol that help to improve the solubility of lipophilic drugs and aid in disrupting the SC. Therefore, the delivery of drugs into the deep dermal layers or even into the systemic circulation is possible [81,82]. When compared to classic liposomes, ethosomes are more stable and achieved superior antifungal activity [83].

Invasomes represent vesicular carriers for enhanced skin delivery. They are composed of unsaturated phospholipids, small amounts of ethanol, terpenes, and water. Different penetration studies performed in vitro in human skin were represented in order to show the penetration-enhancing ability of invasomes. They are characterized by elasticity and deformability, which favors penetration across skin layers [27,28,29,84].

## 4. Conclusions

Nowadays, phytochemicals like terpenes and terpenoids have promising potential to prevent and treat different types of diseases. Although several terpenes are considered GRAS, few studies investigated the safety of these compounds in direct topical application. Their poor water solubility, stability, and bioavailability, as well as other side effects (e.g., irritant index), have limited their clinical application. New drug delivery systems based on lipids represent an encouraging approach for topical applications of terpenes. These systems are attractive, non-invasive, and especially beneficial for patients that are not viable for surgery or highly intensive non-specific systemic therapies. Encapsulation of terpenes in lipid-based nanocarriers is widely described in the literature as an approach providing protection against environmental factors that can cause chemical degradation and volatilization of these compounds. Moreover, nanostructured lipid systems allow controlled drug release and enable the passage of the bioactive compounds through biological barriers making them ideal candidates for topical applications. Finally, terpenes and lipid-based nanosystems represent a sustainable alternative in pharmaceuticals, giving increasing importance to greener chemistry.

## Figures and Tables

**Table 1:** Terpenes as skin permeation enhancers encapsulated in nanostructured lipid systems.

Terpenes	Nanosystem	Administration Route	Experimental Model	Reference
**Limonene, citral, cineole**	Invasomes	Transdermal	In vitro (abdominal human skin)	[27,28]
**β-citronellene**	Invasomes	Transdermal	In vivo (rat skin)	[30,31]
**Limonene, cineole, fenchone, citral**	Invasomes	Cutaneous	In vivo (rat skin)	[32]
**Limonene**	Liposomes	Transdermal	In vitro (porcine skin)	[33]
**Limonene**	PEGylated liposomes	Transdermal	In vitro (porcine skin)	[34,35]
**Limonene**	Nanoemulsion	Transdermal	In vitro (abdominal human skin)	[36]
**Eucalyptol**	Nanoemulsion	Transfollicular		[37]
**Eucalyptol and pinene**	Nanoemulsion	Transdermal	In vivo	[38]
**Limonene and 1,8-cineole**	SLN, NLC, Nanoemulsion	Cutaneous	In vitro	[9]

**Table 2:** Terpenes as bioactive compounds encapsulated in lipid-based nanosystems for anti-inflammatory treatment.

Terpenes/Terpenoids	Nanosystem	Administration Route	Activity	Reference
**Thymol**	SLN	Cutaneous	Anti-inflammatory	[11]
**Astragaloside IV**	SLN	Cutaneous	Wound healing	[43]
**Triptolide**	SLN	Cutaneous	Anti-inflammatory	[44]
**Ursolic acid**	NLC	Cutaneous	Antiarthritic	[45]
**Forskolin**	NLC	Transdermal	Photoprotector	[46]
**Hurpezine A**	SLN, NLC, Microemulsion	Transdermal	Alzheimer’s treatment	[47]
**Triptolide**	Nanoemulsion	Percutaneous	Anti-inflammatory, analgesic	[48]
**Safranal**	Nanoemulsion	Nasal	Cerebral ischemia treatment	[49]
**Madecassoside**	Liposomes	Cutaneous	Wound healing, psoriasis	[50]
**Citral**	Liposomes	Transdermal	Anti-inflammatory, antifungal	[51]
**Thymol, menthol, camphor and cineol**	Invasomes	Transdermal	Anti-inflammatory, bacterial infections e.g., MRSA	[52]

**Table 3:** Terpenes as anticancer bioactive compounds encapsulated in lipid based nanosystems.

Terpenes/Terpenoids	Nanosystem	Administration Route	Activity	Reference
**Paclitaxel**	SLN	Cutaneous	Antineoplastic	[13]
**Tripterine**	NLC	Cutaneous	Antimelanoma	[62]
**Betulin**	Nanoemulsion	Cutaneous	Anti-carcinogenic	[63]
**Ursolic acid**	Nano lipid vesicles	Nasal	Antineoplastic	[64]
**Tripterine**	Phytosomes	Oral/Buccal	Antineoplastic	[65]
